# The effect of preoperative biliary drainage on postoperative complications of pancreaticoduodenectomy: a triple center retrospective study

**DOI:** 10.1186/s12893-022-01853-z

**Published:** 2022-11-18

**Authors:** Niloufar Bineshfar, Nasser Malekpour Alamdari, Tayebeh Rostami, Alireza Mirahmadi, Adel Zeinalpour

**Affiliations:** 1 Ophthalmic Research CenterResearch Institute for Ophthalmology and Vision ScienceShahid Beheshti University of Medical Sciences, Tehran, Iran; 2 Department of General Surgery, Modarres Hospital, Shahid Beheshti University of Medical Sciences, Saadat Abad Blvd., Tehran, 1998734383 Iran; 3 Department of Pathology, School of Medicine, Shahid Beheshti University of Medical Sciences, Tehran, Iran; 4 Bone, Joint and Related Tissues Research Center, Akhtar Orthopedic Hospital, Shahid Beheshti University of Medical Sciences, Tehran, Iran; 5 Clinical Research and Development Center, Modarres Hospital, Shahid Beheshti University of Medical Sciences, Tehran, Iran; 6 Critical Care and Quality Improvement Research Center, Shahid Modarres Hospital, Shahid Beheshti University of Medical Sciences, Tehran, Iran

**Keywords:** Pancreaticoduodenectomy, Preoperative biliary drainage, Biliary stenting, Hyperbilirubinemia, Complications

## Abstract

**Background:**

Biliary obstruction which is a major complication of pancreas and periampullary tumors could result in cholangitis, coagulopathies, gastrointestinal symptoms, and impaired wound healing. Pancreaticoduodenectomy (PD) is still the standard approach for pancreas resection and imposes high risk of morbidity and mortality to patients. To reduce the high risk of PD and address the biliary obstruction, the use of preoperative biliary stenting was increased. However, available literature doubts its efficiency.

**Methods:**

A total of 147 patients who underwent PD between September 2012, and February 2022, at three medical centers were identified. Patients were grouped based on biliary stent placement. Non-jaundiced patients with and without preoperative biliary drainage (PBD) were compared.

**Results:**

The incidence of overall complications (34.2% versus 45.8%) and mortality (17.8% versus 24.3%) did not differ in the PBD group compared to the no PBD group. There was no difference in complications and mortality in non-jaundiced patients with and without PBD. Patients with drainage duration of > 30 days experienced more overall complications compared to patients with less than 30 days drainage duration (12 (50.0%) and three (15.8%) patients, respectively, *p*-value = 0.019).

**Conclusions:**

PBD does not significantly increase the post-operative burden on patients who undergo PD. However, we cannot overlook the financial burden that PBD places on the patient and the healthcare system, as well as the difficulties related to endoscopic retrograde cholangiopancreatography (ERCP). Therefore, biliary stenting should not be routinely practiced in the absence of a valid indication, such as severe jaundice, pruritus, cholangitis, delayed surgery for neoadjuvant treatment, or referral to a tertiary facility.

## Introduction

One of pancreatic and periampullary tumors' main complications is biliary obstruction [1], which could result in cholangitis, coagulopathies, and gastrointestinal symptoms. Cholestasis precipitates bacterial growth within the bile. Bacteria enter circulation after elevated biliary pressure have damaged the hepatic cell and bile microduct barrier. Ensuing reduction in hepatic blood circulation impairs liver metabolic and synthetic function, which in turn results in a chain of events including oxidative stress, decreased plasma albumin, impaired coagulation cascade, and immune system disturbances. Due to the bile salts deficiency, gut normal flora grows excessively, and subsequent intestinal mucosal barrier disruption causes bacterial translocation, and increased endotoxin concentration which ultimately impairs wound healing [2].

Pancreaticoduodenectomy (PD) which is the standard procedure for resecting pancreatic and periampullary tumors, is associated with high mortality and morbidity [3, 4]. According to the above-mentioned reasons, hyperbilirubinemia was hypothesized to be a determining factor in PD outcome. Thus, it was believed that addressing biliary obstruction with preoperative biliary drainage (PBD) would reduce the post-operative complications [5].

In the initial studies, performing PBD yielded promising results. PBD can be accomplished by biliary stenting using endoscopic retrograde cholangiopancreatography (ERCP) or by placement of a percutaneous transhepatic catheter (PTC) [6]. However, given the preoperative complications of PBD, the benefits of this method have been doubted. Throughout the last decade numerous studies have published to address the diversity of outcomes and to reach an agreement. Limited studies have stated favorable effects of PBD on postop outcome in selected patients [7–9], while others report equivalent or even adverse effects of PBD on postoperative complications [10–12]. Latest guidelines suggest selective use of PBD in the following circumstances: cholangitis, neoadjuvant therapy, delayed surgery, and bilirubin level of ≥ 15 mg/dL [13].

Recent studies are coming to an agreement that performing PBD neither improves nor harms the outcome of PD. However, many studies are single-centered, and there is limited evidence from developing countries where patients have difficulty accessing a pancreatic surgeon. Furthermore, decisive variables, such as duration between onset of symptoms and operation, and drainage duration, have not been fully addressed. We have conducted this study to report the result of PBD in an Iranian population.

## Methods

### Study design

This is a retrospective study of patients who underwent PD between September 2012, and February 2022, at Shahid Modarres, Taleghani, and Shohada medical centers in Tehran, Iran. All three hospitals are university hospitals with Taleghani being a medium-volume center for pancreatic surgery (11–19 annually performed PDs) [14]. The participants were identified by extensive review of medical records.

Inclusion criteria were patients undergoing PD with periampullary neoplasm or other benign pathologies. Exclusion criteria were combination of PD with other surgeries and placement of PTC prior to surgery. One patient underwent simultaneous PD and liver transplant, and 23 patients with PTC placement were excluded from the study.

To reduce the effect of biliary obstruction, we further divided the study population into two groups based on biliary obstruction and then assessed variables within the obstructed patients. Biliary obstruction was defined as a subjective report of jaundice prior to admission or a total bilirubin level of > 2 mg/dL. Then patients were grouped based on their PBD situation. Patients who received preoperative biliary stenting were referred to as the PBD group.

### Jaundice management

Most of the patients underwent ERCP and stent placement as part of a gastroenterology assessment before referral to a pancreatic surgeon. Stent size and type (metallic or plastic) were decided by the gastroenterologist. In the event of unsuccessful biliary stenting, PTC was employed. Stent exchange was performed either due to stent disfunction or cholangitis.

### Operation

During the study period, PDs were performed by 12 surgeons who used similar techniques. Prophylactic intravenous antibiotics were administered prior to surgery. Based on the surgeon’s opinion either a classic or a pylorus-preserving Whipple was performed. Following resection, pancreaticojejunostomy, choledochojejunostomy, and gastrojejunostomy were performed sequentially. At the end of the procedure surgical drains were placed. Patients were admitted to the intensive care unit (ICU) for at least 24 h following surgery and were given prophylactic antibiotics.

### Variables

Data of patients’ demographic, symptoms, past medical history, and the American Society of Anesthesiologists (ASA) score were extracted from medical records. Preoperative assessments included preoperative laboratory values within a week prior to surgery, and pathology evaluation. Furthermore, operation duration, estimated blood loss, and postoperative complications were collected.

A weight loss of > 10% in six months was documented. Drainage duration was computed from the date of initial placement until the surgery date. Length of hospital stay was considered the hospitalization days after the surgery. Surgery duration was calculated from the start of anesthesia until the dressing of the surgical wound. Postoperative complications within 30 days after surgery or during hospitalization were recorded. Primary surgical complications were defined as the occurrence of severe postoperative complications (Clavien–Dindo ≥ 3) [15]. Secondary surgical Complications included delayed gastric emptying (DGE), postoperative hemorrhage, postoperative pancreatic fistula (POPF), intraabdominal abscess, and wound infection. Presence of grade C DGE [16], severe postoperative hemorrhage [17], and grade C POPF [18] according to the International Study Group of Pancreatic Society (ISGPS) criteria, were extracted from the medical records. Overall complications were defined as the sum of all complications. Mortality was defined as deaths within 90 days after surgery.

### Ethics

Research Medical Ethics Committee of Shahid Beheshti university of medical sciences reviewed and approved this study (approval number: IR.SBMU.MSP.REC.1398.1008). This study was conducted in accordance with the Declaration of Helsinki and institutional ethics guidelines. For the use of clinical data, written informed consent was obtained from the patients.

### Statistical analysis

Hospitalization was reported as median and interquartile range (IQR). Other continuous variables were presented as mean ± standard deviation (SD). Categorical variables were shown as count (percentage). Kolmogorov–Smirnov test was applied to evaluate continuous variables for normal distribution. Comparisons of parametric and non-parametric continuous variables were executed using Student *t*-test and Mann–Whitney U test, respectively. Categorical variables were analyzed using Chi-square or Fisher’s exact test. Also, the effect of different risk factors on overall morbidity and mortality was evaluated using univariate and multivariate analyses. Risk factors with a *p*-value of less than 0.1 in the univariate analysis, were used for multivariate regression modeling. Data were analyzed with SPSS version 26 (IBM, Armonk, NY, USA). A *p*-value of < 0.05 was considered statistically significant.

## Results

Overall, 147 patients were included in the study with mean age of 57.4 ± 12.3 (range 20–87, median = 58.0) years. Ninety-six (65.3%) patients were male. The most prevalent symptoms were jaundice, abdominal pain, and weight loss, respectively. Eight patients undergoing PBD experienced fever, and two of them developed cholangitis prior surgery. Hypertension and diabetes were most prevalent comorbidities. Detailed description of patients characteristics is shown in Table1.

**Table 1:** Detailed patients characteristics

Characteristics	Total (n = 147)	PBD group (n = 73)	No PBD group (n = 74)	*p*-value
Age, mean ± SD, year	57.4 ± 12.3	57.7 ± 10.8	57.0 ± 13.8	0.862
Male gender, no. (%)	96 (65.3)	52 (71.2)	44 (59.5)	0.134
Symptoms				
Duration, mean ± SD, days	106 ± 120	134 ± 148	76 ± 71	0.050
Jaundice, no. (%)	92 (82.1)	60 (98.4)	32 (62.7)	** < 0.001**
Abdominal pain, no. (%)	63 (64.9)	34 (68.0)	29 (61.7)	0.516
Weight loss, no. (%)	41 (43.6)	22 (44.9)	19 (42.2)	0.794
Malaise, no. (%)	28 (30.4)	17 (36.2)	11 (24.4)	0.222
Nausea/vomiting, no. (%)	17 (18.1)	10 (20.4)	7 (15.6)	0.541
Fever, no. (%)	9 (8.7)	8 (15.1)	1 (2.0)	**0.032**
Medical history				
Diabetes, no. (%)	26 (18.7)	15 (21.4)	11 (15.9)	0.407
Hypertension, no. (%)	36 (26.1)	20 (29.0)	16 (23.2)	0.438
Coronary artery disease, no. (%)	15 (11.0)	8 (11.8)	7 (10.3)	0.782
Thyroid disease, no. (%)	7 (5.1)	2 (2.9)	5 (7.4)	0.441
Chronic renal failure, no. (%)	0	0	0	–
Liver disease, no. (%)	0	0	0	–
Neoadjuvant therapy, no. (%)	8 (5.7)	6 (8.5)	2 (2.9)	0.275
ASA score > 2	49 (35.8)	26 (36.6)	23 (34.8)	0.900
Preop lab				
WBC, mean ± SD, × 10^9^ cells/l	7.5 ± 1.2	6.8 ± 2.5	8.4 ± 3.0	**0.020**
Lymphopenia, no. (%)	10 (14.3)	3 (8.1)	7 (21.2)	0.173
Total bilirubin, mean ± SD, mg/dl	6.9 ± 8.5	3.45 ± 4.5	10.9 ± 10.2	** < 0.001**
Total bilirubin > 10 mg/dl, no. (%)	31 (25.4)	4 (6.1)	27 (48.2)	** < 0.001**
Alkaline phosphatase, mean ± SD, U/l	701.6 ± 630.0	492.5 ± 403.1	914.7 ± 743.0	** < 0.001**
Albumin, mean ± SD, g/dl	3.8 ± 0.5	3.8 ± 0.6	3.7 ± 0.5	0.510
Total protein, mean ± SD,	6.4 ± 0.9	6.3 ± 0.9	6.5 ± 0.9	0.454
Creatinine, mean ± SD, mg/dl	0.88 ± 0.29	0.92 ± 0.26	0.86 ± 0.30	0.120
Histopathology				
Histology				**0.034**
Pancreatic adenocarcinoma, no. (%)	42 (34.1)	25 (39.7)	17 (28.3)	–
Ampullary cancer, no. (%)	41 (33.3)	26 (41.3)	15 (25.0)	–
Duodenal cancer, no. (%)	10 (8.1)	5 (7.9)	5 (8.3)	–
Neuroendocrine tumor, no. (%)	7 (5.7)	1 (1.6)	6 (10.0)	–
Distal cholangiocarcinoma, no. (%)	6 (4.9)	2 (3.2)	4 (6.7)	–
Other, no. (%)	12 (9.8)	3 (4.8)	9 (15.0)	–
Unknown, no. (%)	5 (4.1)	1 (1.6)	4 (6.7)	–
Tumor size, mean ± SD, mm	35 ± 19	29 ± 16	41 ± 20	**0.005**
Operation				
Harvested LNs, mean ± SD	9.7 ± 7.0	9.5 ± 6.5	9.9 ± 7.5	0.814
Estimated blood loss, mean ± SD, ml	951 ± 499	1003 ± 538	894 ± 453	0.458
Surgery duration, mean ± SD, minutes	464 ± 151	477 ± 147	450 ± 155	0.486

Bold *p*-values represent *p*-values <0.05

*WBC* white blood cells, *LN* lymph node

Seventy-three patients received preoperative biliary stenting, among them five (7.2%) patients received stent exchange once, and one (1.4%) patient received stent exchange twice. The mean of drainage period was 96 ± 121 (range 5–440, median = 41.5) days. Also, 24 (56%) patients had drainage duration of more than 30 days. Plastic stent was used for 32 (84.2%) patients, and metallic for six (15.8%).

### All patients

Concerning patient demographics, the only significant differences between PBD and no PBD groups were prevalence of jaundice (*p*-value < 0.001) and fever (*p*-value = 0.03) before surgery. Also, no PBD group had significantly higher levels of WBC (*p*-value = 0.02), bilirubin (*p*-value < 0.001), and alkaline phosphatase (*p*-value < 0.001) compared to PBD group.

### Patients with biliary obstruction

After excluding non-obstructed patients, there was no significant difference in symptoms and medical history between PBD and no PBD groups. the mean duration of symptoms was considerably longer in PBD group (136 ± 149 versus 67 ± 50). Patients undergoing PBD had lower total bilirubin and alkaline phosphatase levels (*p*-value < 0.001) before surgery (Table2).

**Table 2:** Detailed characteristics of patients with biliary obstruction

Characteristics	Total (n = 120)	PBD group (n = 72)	No PBD group (n = 48)	*p*-value
Age, mean ± SD, year	56.8 ± 11.9	57.4 ± 10.6	56.0 ± 13.8	0.641
Male gender, no. (%)	83 (69.2)	51 (70.8)	32 (66.7)	0.629
Symptoms				
Duration, mean ± SD, days	109 ± 125	136 ± 149	67 ± 50	**0.031**
Jaundice, no. (%)	92 (98.9)	60 (100)	32 (97.0)	0.355
Abdominal pain, no. (%)	51 (64.6)	33 (67.3)	18 (60.0)	0.508
Weight loss, no. (%)	37 (48.7)	22 (45.8)	15 (53.6)	0.515
Malaise, no. (%)	25 (33.8)	17 (37.0)	8 (28.6)	0.460
Nausea/ vomiting, no. (%)	16 (20.8)	10 (20.8)	6 (20.7)	0.988
Fever, no. (%)	9 (10.7)	8 (15.4)	1 (3.1)	0.143
Medical history				
Diabetes, no. (%)	24 (21.1)	15 (21.7)	9 (20.0)	0.824
Hypertension, no. (%)	31 (27.4)	20 (29.4)	11 (24.4)	0.562
Coronary artery disease, no. (%)	13 (11.7)	8 (11.9)	5 (11.4)	0.926
Thyroid disease, no. (%)	5 (4.5)	2 (3.0)	3 (6.8)	0.383
Chronic renal failure, no. (%)	0	0	0	–
Liver disease, no. (%)	0	0	0	–
Neoadjuvant therapy, no. (%)	7 (6.1)	6 (8.6)	1 (2.2)	0.275
ASA score > 2	43 (39.1)	26 (38.2)	17 (40.5)	0.815
Preop lab				
WBC, mean ± SD, × 10^9^ cells/l	7.3 ± 2.7	6.8 ± 2.6	8.1 ± 2.8	0.078
Lymphopenia, no. (%)	7 (11.9)	3 (8.1)	4 (18.2)	0.407
Total bilirubin, mean ± SD, mg/dl	7.9 ± 8.8	3.5 ± 4.6	14.9 ± 9.4	** < 0.001**
Total bilirubin > 10 mg/dl, No. (%)	31 (29.5)	4 (6.2)	27 (67.5)	** < 0.001**
Alkaline phosphatase, mean ± SD, U/l	761 ± 641	487 ± 405	1139 ± 716	** < 0.001**
Albumin, mean ± SD, g/dl	3.8 ± 0.5	3.8 ± 0.6	3.7 ± 0.5	0.473
Total protein, mean ± SD, g/dl	6.4 ± 0.9	6.3 ± 0.9	6.5 ± 0.8	0.588
Creatinine, mean ± SD, mg/dl	0.87 ± 0.23	0.92 ± 0.26	0.79 ± 0.15	**0.044**
Histopathology				
Histology				0.150
Pancreatic adenocarcinoma, no. (%)	39 (39.0)	25 (40.3)	14 (36.8)	–
Ampullary cancer, no. (%)	33 (33.0)	25 (40.3)	8 (21.1)	–
Duodenal cancer, no. (%)	10 (10.0)	5 (8.1)	5 (13.2)	–
Neuroendocrine tumor, no. (%)	4 (4.0)	1 (1.6)	3 (7.9)	–
Distal cholangiocarcinoma, no. (%)	6 (6.0)	2 (3.2)	4 (10.5)	–
Other, no. (%)	5 (5.0)	3 (4.8)	2 (5.3)	–
Unknown, no. (%)	3 (3.0)	1 (1.6)	2 (5.3)	–
Tumor size, mean ± SD, mm	31 ± 15	29 ± 16	35 ± 12	0.063
Operation				
Harvested LNs, mean ± SD	9.8 ± 6.9	9.7 ± 6.4	10.0 ± 7.7	0.820
Estimated blood loss, mean ± SD, ml	978 ± 509	1012 ± 540	921 ± 457	0.577
Surgery duration, mean ± SD, minutes	474 ± 143	480 ± 147	464 ± 139	0.925

Bold *p*-values represent *p*-values <0.05

*WBC* white blood cells, *LN* lymph node

### Postoperative outcome

Overall postoperative morbidity and mortality was 40.7% and 21.1%, respectively. Primary surgical complications rate was 15.0% and wound infection was the most common secondary surgical complication with 14.8% incidence. Other common complications were hemorrhage, intraabdominal abscess, POPF, and DGE, respectively.

There was no difference in post-operative morbidity and mortality between PBD and no PBD group (Table 3).

**Table 3:** Postoperative outcomes

Postop outcomes	Total (n = 147)	PBD group (n = 73)	No PBD group (n = 74)	*p*-value
Hospitalization, median (IQR), day	12 (8–21)	12 (9–18)	12 (7–23)	0.696
Primary surgical complications, no. (%)	22 (15.0)	9 (12.3)	13 (17.6)	0.489
Wound infection, no. (%)	21 (14.8)	9 (12.7)	12 (16.9)	0.478
Hemorrhage, no. (%)	13 (9.2)	6 (8.6)	7 (9.9)	0.792
Intraabdominal abscess, no. (%)	12 (8.5)	8 (11.3)	4 (5.6)	0.228
Grade C POPF, no. (%)	9 (6.3)	4 (5.6)	5 (7.0)	1.000
Grade C DGE, no. (%)	8 (5.6)	6 (8.5)	2 (2.8)	0.275
Overall morbidity, no. (%)	58 (40.7)	25 (34.2)	33 (45.8)	0.177
Mortality, no. (%)	31 (21.1)	13 (17.8)	18 (24.3)	0.333

*IQR* interquartile range, *POPF* postoperative pancreatic fistula, *DGE* delayed gastric emptying

Bilirubin level of > 15 mg/dl was correlated with higher overall complications (63.6% versus 33.0%, *p*-value = 0.008).

Surgery duration was significantly longer in patients with wound infection (*p*-value = 0.002 m), intraabdominal abscess (*p*-value = 0.003 m), DGE (*p*-value = 0.003 m), secondary complications (*p*-value = 0.001 m), overall complications (*p*-value = 0.005 m).

### Postoperative outcome in patients with biliary obstruction

Post-operative morbidity and mortality were similar between PBD and no PBD group in patients with biliary obstruction (Table 4).

**Table 4:** Postoperative outcomes of patients with biliary obstruction

Postop outcomes	Total (n = 120)	PBD group (n = 72)	No PBD group (n = 48)	*p*-value
Hospitalization, median (IQR), day	12 (8–21)	12 (9–18)	12 (7–23)	0.776
Primary surgical complications, no. (%)	22 (15.0)	9 (12.5)	10 (20.8)	0.221
Wound Infection, no. (%)	21 (14.8)	9 (12.9)	8 (17.4)	0.499
Hemorrhage, no. (%)	13 (9.2)	6 (8.7)	6 (13)	0.539
Intraabdominal abscess, no. (%)	12 (8.5)	8 (11.4)	2 (4.3)	0.311
Grade C POPF, no. (%)	9 (6.3)	4 (5.7)	3 (6.5)	1.000
Delayed gastric emptying, no. (%)	8 (5.6)	6 (8.6)	1 (2.2)	0.241
Overall morbidity, no. (%)	58 (40.7)	25 (34.7)	24 (51.1)	0.077
Mortality, no. (%)	31 (21.1)	13 (18.1)	13 (27.1)	0.240

*IQR* interquartile range, *POPF* postoperative pancreatic fistula

Symptom duration was not associated with increased risk of mortality, overall morbidity, primary surgical complications, or any secondary surgical complications (*p*-value > 0.05).

### PBD subgroup analysis

In the PBD group, bilirubin level of > 10 mg/dl was associated with higher secondary surgical complications (75.0% versus 21.0%, *p*-value = 0.041). In the no PBD group, mortality rate was higher in patients with a bilirubin level of greater than 15 mg/dl (40.0% versus 11.1%, *p*-value = 0.018).

Drainage duration was not associated with increase in primary surgical complications, wound infection, hemorrhage, intraabdominal abscess, POPF, DGE, mortality, or hospitalization (*p*-value > 0.05 m). However, patients with drainage duration of > 30 days significantly experienced more overall complications compared to patients with less than 30 days drainage duration (12 (50.0%) versus three (15.8%) patients, respectively, *p*-value = 0.019).

Patients with plastic and metallic stent did not differ in any complications, mortality, or hospitalization (*p*-value > 0.05). Patients with stent exchange were comparable to patient without exchange regarding complications, mortality, and hospitalization (*p*-value > 0.05).

### Univariate and multivariate analyses in all patients

Univariate and multivariate analyses were conducted to detect independent predictors of outcomes in all patients (Table 5). In univariate analysis, total bilirubin, estimated blood loss and surgery duration affected overall morbidity; however, these variables did not significantly increase or decrease the probability of overall morbidity. In addition, no predictors were identifiable in the multivariate analysis of overall morbidity. In univariate and multivariate analyses of mortality, no risk factors were identified.

**Table 5:** Univariate and multivariate analyses of risk factors for overall morbidity and mortality in all patients (n = 147)

Characteristics	Overall morbidity				Mortality			
	Univariate analysis		Multivariate analysis		Univariate analysis		Multivariate analysis	
	OR (95% CI)	*p*	OR (95% CI)	*p*	OR (95% CI)	*p*	OR (95% CI)	*p*
Age	1.004 (0.976–1.032)	0.796			1.028 (0.993–1.064)	0.117		
Male gender	1.000 (0.497–2.012)	1.000			0.957 (0.418–2.193)	0.917		
Stent placement	1.625 (0.832–3.174)	0.156			0.674 (0.303–1.502)	0.334		
Symptoms								
Duration	0.999 (0.996–1.003)	0.623			0.999 (0.995–1.004)	0.789		
Jaundice	0.447 (0.137–1.458)	0.182			2.500 (0.535–11.692)	0.244		
Abdominal pain	1.028 (0.421–2.512)	0.951			1.365 (0.437–4.261)	0.592		
Weight loss	0.834 (0.349–1.995)	0.683			1.185 (0.413–3.400)	0.752		
Malaise	0.704 (0.274–1.814)	0.468			2.037 (0.672–6.171)	0.208		
Nausea/vomiting	4.286 (0.913–20.126)	0.065	4.726 (0.534–41.834)	0.163	0.238 (0.029–1.934)	0.179		
Fever	0.595 (0.149–2.377)	0.463			2.265 (0.514–9.970)	0.280		
Medical history								
Diabetes	0.838 (0.352–1.991)	0.688			0.674 (0.212–2.144)	0.504		
Hypertension	0.610 (0.282–1.317)	0.208			1.556 (0.626–3.865)	0.341		
Coronary artery disease	1.270 (0.409–3.948)	0.679			0.622 (0.131–2.942)	0.549		
Thyroid disease	0.817 (0.175–3.804)	0.796			1.750 (0.320–9.567)	0.518		
Neoadjuvant therapy	0.650 (0.156–2.714)	0.555			2.446 (0.549–10.900)	0.241		
ASA score > 2	0.839 (0.411–1.713)	0.631			1.670 (0.726–3.842)	0.228		
Preop lab								
WBC	1.000 (1.000–1.000)	0.306			1.000 (1.000–1.000)	0.122		
Lymphopenia	2.667 (0.521–13.655)	0.239			1.250 (0.230–6.786)	0.796		
Total bilirubin	0.952 (0.911–0.994)	**0.026**	0.966 (0.907–1.030)	0.294	1.043 (0.994–1.094)	0.089	1.072 (0.981–1.171)	0.126
Alkaline phosphatase	1.000 (0.999–1.000)	0.408			1.000 (0.999–1.001)	0.941		
Albumin	1.574 (0.661–3.748)	0.305			0.792 (0.291–2.154)	0.648		
Total protein	0.809 (0.387–1.691)	0.574			2.979 (0.900–9.865)	0.074	3.191 (0.890–11.449)	0.075
Creatinine	1.084 (0.243–4.833)	0.916			2.372 (0.452–12.452)	0.307		
Histopathology								
Tumor size	1.304 (0.933–1.822)	0.120			0.798 (0.535–1.188)	0.266		
Operation								
Harvested LNs	0.998 (0.940–1.059)	0.938			1.026 (0.958–1.100)	0.461		
Estimated blood loss	0.999 (0.998–1.000)	**0.015**	0.999 (0.996–1.001)	0.346	1.001 (1.000–1.002)	0.105		
Surgery duration	0.997 (0.994–0.999)	**0.008**	1.001 (0.993–1.009)	0.783	1.002 (1.000–1.005)	0.106		

Bold *p*-values represent *p*-values <0.05

*WBC* white blood cells, *LN* lymph node

### Univariate and multivariate analyses in patients with biliary obstruction

Univariate and multivariate analyses were carried out to identify independent predictors of outcomes in obstructed patients (Table 6). Although stent placement, nausea and vomiting, total bilirubin, estimated blood loss and surgery duration were selected for multivariate analysis of overall morbidity, these risk factors did not significantly change the possibility of overall morbidity. In univariate and multivariate analyses of morbidity, no risk factor significantly impacted the outcomes.

**Table 6:** Univariate and multivariate analyses of risk factors for overall morbidity and mortality in patients with biliary obstruction (n = 120)

Characteristics	Overall morbidity				Mortality			
	Univariate analysis		Multivariate analysis		Univariate analysis		Multivariate analysis	
	OR (95% CI)	*p*	OR (95% CI)	*p*	OR (95% CI)	*p*	OR (95% CI)	*p*
Age	1.015 (0.983–1.048)	0.357			1.018 (0.980–1.058)	0.349		
Male gender	1.029 (0.465–2.277)	0.943			0.801 (0.319–2.013)	0.637		
Stent placement	1.962 (0.927–4.153)	0.078	0.229 (0.050–1.037)	0.056	0.593 (0.247–1.423)	0.242		
Symptoms								
Duration	0.999 (0.995–1.002)	0.419			1.000 (0.995–1.004)	0.956		
Abdominal pain	0.798 (0.301–2.116)	0.650			1.650 (0.472–5.766)	0.433		
Weight loss	1.034 (0.404–2.647)	0.945			0.904 (0.292–2.804)	0.862		
Malaise	0.862 (0.313–2.370)	0.773			1.618 (0.492–5.320)	0.428		
Nausea/vomiting	4.861 (1.014–23.296)	**0.048**	5.215 (0.522–52.122)	0.160	0.224 (0.027–1.847)	0.164		
Fever	0.703 (0.174–2.842)	0.621			2.000 (0.448–8.936)	0.364		
Medical history								
Diabetes	0.933 (0.374–2.329)	0.882			0.700 (0.214–2.285)	0.555		
Hypertension	0.513 (0.222–1.185)	0.118			1.554 (0.583–4.140)	0.378		
Coronary artery disease	1.552 (0.447–5.388)	0.489			0.306 (0.038–2.486)	0.268		
Thyroid disease	0.984 (0.158–6.143)	0.987			2.867 (0.449–18.308)	0.266		
Neoadjuvant therapy	0.516 (0.156–2.714)	0.401			2.932 (0.611–14.071)	0.179		
ASA score > 2	1.167 (0.535–2.544)	0.697			1.051 (0.422–2.613)	0.916		
Preop lab								
WBC	1.000 (1.000–1.000)	0.501			1.000 (1.000–1.000)	0.343		
Lymphopenia	4.065 (0.456–36.255)	0.209			0.796 (0.085–7.447)	0.842		
Total bilirubin	0.953 (0.911–0.998)	**0.039**	1.013 (0.934–1.097)	0.759	1.039 (0.998–1.093)	0.134		
Alkaline phosphatase	1.000 (0.999–1.000)	0.608			1.000 (0.999–1.001)	0.908		
Albumin	1.514 (0.588–3.900)	0.391			1.231 (0.421–3.604)	0.704		
Total protein	0.923 (0.418–2.036)	0.843			4.679 (0.952–23.004)	0.058	3.931 (0.058–266.177)	0.524
Creatinine	4.730 (0.475–47.094)	0.185			0.524 (0.034–8.110)	0.644		
Histopathology								
Tumor size	1.286 (0.843–1.964)	0.243			0.826 (0.507–1.348)	0.445		
Operation								
Harvested LNs	0.994 (0.931–1.062)	0.861			1.033 (0.959–1.112)	0.391		
Estimated blood loss	0.999 (0.998–1.000)	**0.023**	0.999 (0.996–1.002)	0.369	1.001 (1.000–1.002)	0.053	1.004 (0.990–1.017)	0.620
Surgery duration	0.997 (0.994–1.000)	**0.023**	1.000 (0.991–1.009)	0.977	1.004 (1.000–1.007)	**0.029**	0.995 (0.950–1.042)	0.830

Bold *p*-values represent *p*-values <0.05

*WBC* white blood cells, *LN* lymph node

## Discussion

Obstructive, painless jaundice is still the most typical scenario of periampullary malignancies. Before being referred to a pancreatic surgeon, most patients had already undergone biliary stenting during an upper endoscopy as part of a malignancy workup. Attempts to alleviate blockage with regular preoperative biliary drainage (PBD) have failed to show an advantage in patient outcomes, although previous research had previously revealed that impaired hepatic function and nutritional state are induced by cholestasis.

In this retrospective study, the incidence of postoperative complications of PD was compared between patients receiving endoscopic retrograde biliary drainage (ERBD) and patients without PBD. Regarding postoperative complications, there was no association between stent placement and incidence of wound infection, hemorrhage, intraabdominal abscess, POPF, and DGE. Primary, secondary, and overall surgical complications, as well as mortality, were not significantly different between ERBD and no PBD groups.

Despite a growing body of evidence showing no advantage for routine PBD, in practice, PBD continues to be widely performed. Several studies have even shown the inferiority of PBD compared to surgery first approach. Fang et al. in a meta-analysis study, showed that the relative risk of overall complications is higher in the ERBD group compared to people without ERBD (rate ratio 1.66; 95% confidence interval (CI):1.28–2.16; *p*-value = 0.001) [19]. Similarly, PBD using stent placement was associated with an increased risk of overall complications compared with immediate surgery in a meta-analysis conducted by Scheufele et al. (odds ratio 1.40; CI: 1.14–1.72; *p*-value = 0.002) [20]. However, in our study, the incidence of overall complications was not significantly different in the ERBD group compared to the no PBD group (34.2% versus 45.8%).

Interestingly, in more updated studies, complications tend to be milder and limited to wound infection. A large-scale cohort by Garcia-Ochoa et al. within the American College of Surgeons National Surgical Quality Improvement Program (ACS NSQIP) registry demonstrated that the ERBD group compared to the no ERBD group, had a 55% and 53% risk of postoperative complication, respectively (risk ratio 1.04; CI: 0.97–1.11; *p-*value = 0.23) [21]. Based on a recent cohort by Webra et al., PBD, accomplished either by PTC or ERBD, was shown to have an equivalent risk of developing major morbidity and mortality in patients undergoing PD. However, PBD imposed 40% higher odds of superficial surgical site infection (SSI) to patients with biliary obstruction (odds ratio 1.40; CI: 1.04–1.89; *p*-value = 0.026) [22]. Additionally, some studies report similar results regarding wound infection [20, 23–25]. Nevertheless, in our study, there was no difference in the incidence of wound infection between groups.

Regarding postoperative hemorrhage, intraabdominal abscess, POPF, and DGE, our results were similar to a retrospective study conducted at Massachusetts General Hospital, showing no considerable difference between patients who received ERBD and no PBD patients [23]. Although some studies have reported an increase in postoperative DGE [24, 26], we did not observe any significant difference between ERBD and no PBD groups.

We did not found any difference in postoperative hospitalization between PBD and no PBD groups (median of 12 and 12 days, respectively), which is comparable to the results of Webra et al. [22]. However, one study has reported a shorter length of hospital stay in patients receiving biliary stating [21], and conversely, a randomized trial by van der Gaag et al. demonstrated significantly longer hospitalization in the PBD group [25].

Although operation duration was associated with an increased risk of developing wound infection, intraabdominal abscess, DGE, secondary surgical complications, and overall complications, stent placement did not significantly affect surgery duration.

Based on our study, severe hyperbilirubinemia (bilirubin > 15 mg/dl), regardless of stent placement, was associated with higher overall complications. Furthermore, our study highlights the risk of secondary surgical complications in patients with bilirubin level of > 10 mg/dl in the PBD group due to stent failure. Consistently, in previous studies a bilirubin level of greater than 7.5–15 mg/dl is reported to be associated with higher rates of post operative complications [1, 27–29]. Interestingly a recent meta-analysis revealed that in bilirubin level of > 15 mg/dl, surgery outcomes do not differ in the PBD and no PBD groups [30].

Based on our observation, patients with drainage duration of more than 30 days experienced more overall complications. However, a retrospective study on 304 patients by Scheufele et al. showed that patients with drainage duration of > 4 weeks and < 4 weeks did not differ in survival [31].

Our study did not identify any difference in surgery outcomes between metallic or plastic stent use. This is comparable with the results of a recent meta-analysis by Watanabe et al., which demonstrated no significant difference in stent-related (risk ratio 0.74; CI: 0.32–1.71) and postoperative complications (risk ratio 0.73; CI: 0.45–1.17) [32]. However, the results of a prospective study demonstrated that PBD-related complication rates were higher in patients receiving plastic stents compared to the metallic stent (46% versus 24%) (relative risk of plastic stent use 1.9, CI: 1.1–3.2; *p*-value = 0.011) [33]. Moreover, in a network meta-analysis, Lee et al. revealed that metallic stents have fewer stent-related complications than plastic stents. Whereas the postoperative outcomes were comparable in both groups (odds ratio 0.99; CI:0.65–1.49; *p*-value > 0.05) [34].

The PBD group had a higher prevalence of ampullary and pancreatic tumors, whereas the no PBD group had a higher prevalence of neuroendocrine tumors. The anatomical origins of neuroendocrine tumors and the fact that they are less prone to result in obstructive jaundice might be the reasons for this variation.

The current study had some limitations, including its retrospective nature, limited capacity to detect mild outcomes, lack of access to the patients' medical records prior to stent placement, and inability to diagnose POPF at its initial stage.

## Conclusions

In conclusion, PBD does not significantly increase the post-operative burden on patients who undergo PD. However, we cannot overlook the financial burden that PBD places on the patient and the healthcare system, as well as the difficulties related to ERCP. Therefore, biliary stenting should not be routinely practiced in the absence of a valid indication, such as severe jaundice, pruritus, cholangitis, delayed surgery for neoadjuvant treatment, or referral to a tertiary facility.

## Data Availability

The data that support the findings of this study are available from Shahid Beheshti University of Medical Sciences, but restrictions apply to the availability of these data, which were used under license for the current study, and so are not publicly available. Data are however available from the corresponding author upon reasonable request and with permission of Shahid Beheshti University of Medical Sciences.
